# Prospecting the theragnostic potential of the psycho-neuro-endocrinological perturbation of the gut-brain-immune axis for improving cardiovascular diseases outcomes

**DOI:** 10.3389/fmolb.2023.1330327

**Published:** 2024-01-25

**Authors:** Emilda Judith Ezhil Rajan, Sai Varsaa Alwar, Richa Gulati, Rohan Rajiv, Tridip Mitra, Rajiv Janardhanan

**Affiliations:** ^1^ Department of Clinical Psychology, Faculty of Medicine and Health Sciences, SRM Institute of Science and Technology, Kattankulathur, India; ^2^ Researcher, Division of Medical Research, Faculty of Medical and Health Sciences, SRM IST, Kattankulathur, India; ^3^ Dietrich School of Arts and Sciences, University of Pittsburgh, Pittsburgh, PaA, United States; ^4^ Division of Medical Research, Faculty of Medicine and Health Sciences, SRM Institute of Science and Technology, Kattankulathur, India

**Keywords:** exosomal microRNA, gut-brain immune axis, depression, diabetes, cardiovascular diseases (CVD)

## Abstract

Biological derivatives and their effective influence on psychological parameters are increasingly being deciphered to better understand body-mind perspectives in health. Recent evidence suggests that the gut-brain immune axis is an attractive theragnostic target due to its innate capacity to excite the immune system by activating monocyte exosomes. These exosomes induce spontaneous alterations in the microRNAs within the brain endothelial cells, resulting in an acute inflammatory response with physiological and psychological sequelae, evidenced by anxiety and depression. Exploring the role of the stress models that influence anxiety and depression may reflect on the effect and role of exosomes, shedding light on various physiological responses that explain the contributing factors of cardiovascular disorders. The pathophysiological effects of gut-microbiome dysbiosis are further accentuated by alterations in the glucose metabolism, leading to type 2 diabetes, which is known to be a risk factor for cardiovascular disorders. Understanding the role of exosomes and their implications for cell-to-cell communication, inflammatory responses, and neuronal stress reactions can easily provide insight into the gut-brain immune axis and downstream cardiovascular sequelae.

## 1 Introduction

Recent advances in clinical psychological approaches to addressing medical conditions have gained popularity as marked changes are recorded empirically. The well-known concept of the stress immune system was brought forth by Maier and Watkins (1998) (Segerstrom and Miller, 2004a), which describes the dynamics of how immunological changes are widely interactive with the psychological stress experienced by an individual (Morey JN. et al., 2015). The concept of stress is broad and comprises two major components: the stressor (a demanding situation) and stress response (the physiological and psychological response) during acute stress reactions; cell mobilization changes in the body when responding to potential harm that can lead to injury and infection caused by the flight and fight response of the stress (Segerstrom and Miller, 2004b). The primary objective of this Review is to explore the role of the stress model governing the neurophysiology and bio-behavioral functions that may perturb the gut-brain immune axis. The last section of our Review focuses on exploiting the potential of exosomal microRNA signatures as theragnostic targets for bio-behavioral anomalies such as cardiovascular disorders, in which depression is known to be correlated with the gut-brain immune axis (Segerstrom and Miller, 2004b).

## 2 Stress and immune functions and the inflammatory response

**FIGURE 1:**
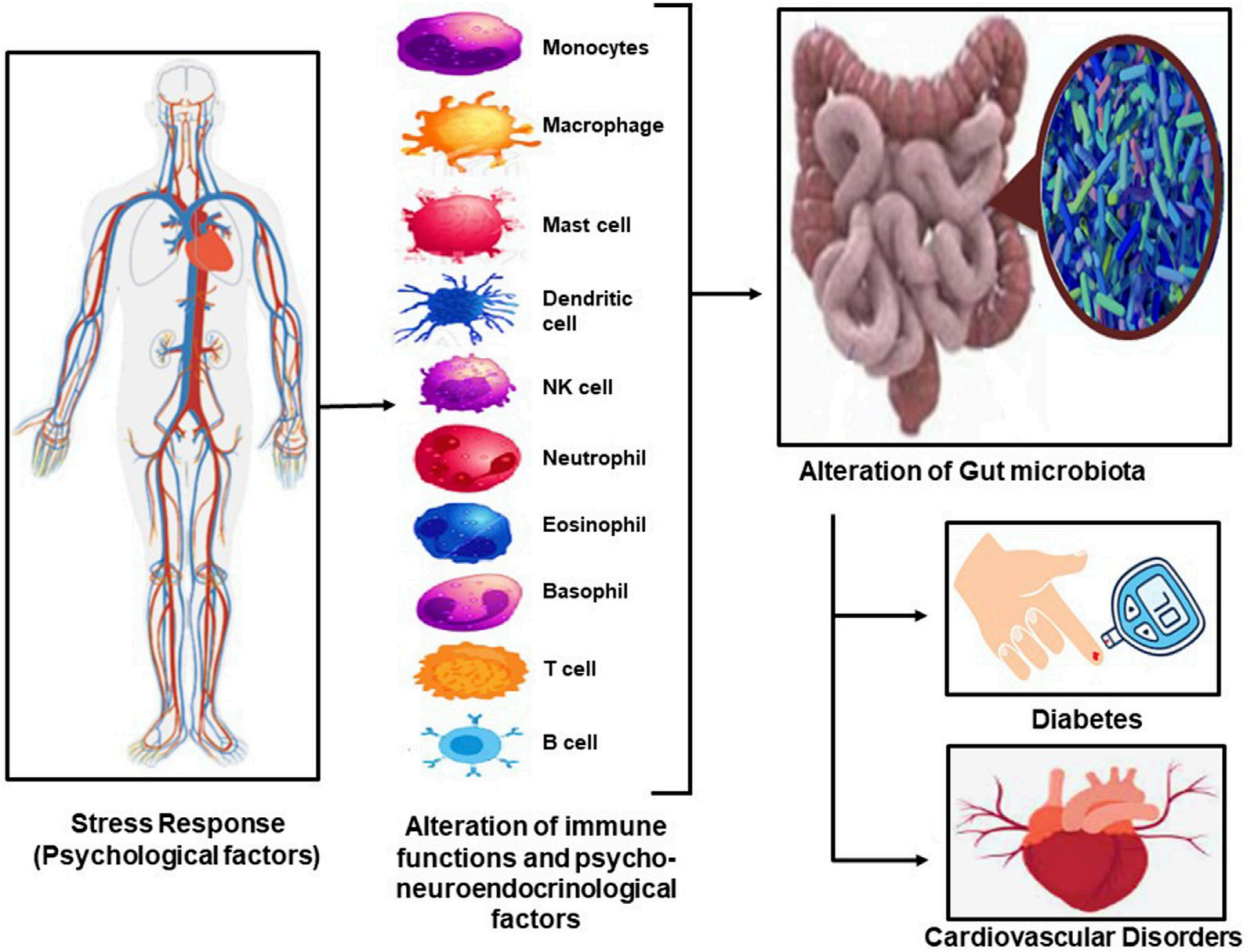
The stress response in our body leads to the excitation of the immune system and the psycho-neuro-endocrinological illustrates leating to alteration in gut microbiota, which results in diabetes and CVD and the interlinkage between the two.

Psychological stress has been implicated in altered immune functioning. Stress induces chronic immune activation and altered health outcomes that lead to exacerbated symptoms of both physical and psychological illnesses (Morey J. N. et al., 2015). Exposure to a variety of acute stressors facilitates the basic properties and immunological functions of exosomes and the efficacy of innate immune responses, providing supporting roles for stress-modified exosomes in regulating the innate immune response, potentially enabling long-distance cellular communication (Beninson and Fleshner, 2014). The role of stress and its relationship to the immune system can further be extended to the neuroendocrine system (Figure 1). It has been shown that the inception of brain immune crosstalk is affected by one’s mental status and psychosocial factors, leading to the concept of psycho-neuro-endocrine-immunology, integrating both psychological and biological science (Bottaccioli et al., 2019). The effect of prolonged stress on neurological functioning and neural structure is a well-established phenomenon. Factors such as a reduction in grey matter, changes in neural structure, neural biochemistry, and brain-derived neurotrophic factors, disruption of the tissue plasminogen activator, and alterations to cell surface molecules lead to structural and functional changes in the brain based on the extent of stress exposure (McEwen et al., 2016).

### 2.1 Immune functions and CVS

Given these extended factors, the excitation of the immune system leads to the activation of monocyte exosomes that can alter the microRNAs in the brain. Furthermore, endothelial cells cause inflammatory responses, resulting in physiological and psychological changes in humans (Dalvi et al., 2017). Exosomes play a critical role in the progression and regulation of cardiovascular diseases, primarily through the transport and exchange of signal molecules (Dalvi et al., 2017). The regulation factor of physiological processes by exosomes and the therapeutic potential is of immense importance in understanding cardiovascular diseases mechanisms (Barile et al., 2017). Addressing exosome changes can highlight their effect on human physiology, which can also be viewed from a preventive angle of addressing stress influencing immune response. This is directly expressed by altering exosome conditions, leading to inflammatory reactions that can affect cardiovascular progression (Wirtz and von Känel, 2017; Salama et al., 2023).

The relationship between the casualties of cardiovascular progression and its comorbid ailments that contribute to the disease is furthered by behavioral and psychosocial constraints placed on the individual. This is well observed in lifestyle diseases; notably, diabetes has emerged as a growing challenge, with childhood diabetes linked to a higher risk of developing CVD (Salama et al., 2023). Interfered with both behavioral approaches and medical/biological intervention, the behavioral process of educating one about CVD and Diabetes has shown greater efficacy, though their behavioral maintenance is yet to be studied (Parisi et al., 2023). The biological or medical perspectives have greatly bagged on noting exosomal behavior specific to its production, regulation of secretion or uptake that enables one to understand pathological mechanisms and devise methods of diagnosing and treating diabetes (Sufianov et al., 2023). The process of developing an effective methodology for a lifestyle disorder will flourish with behavioral processes that can be conditioned or modified based on a keen understanding of the individual psychological changes. This starts at a cellular level.

## 3 Gut-immune-axis and neuroendocrine system

**FIGURE 2:**
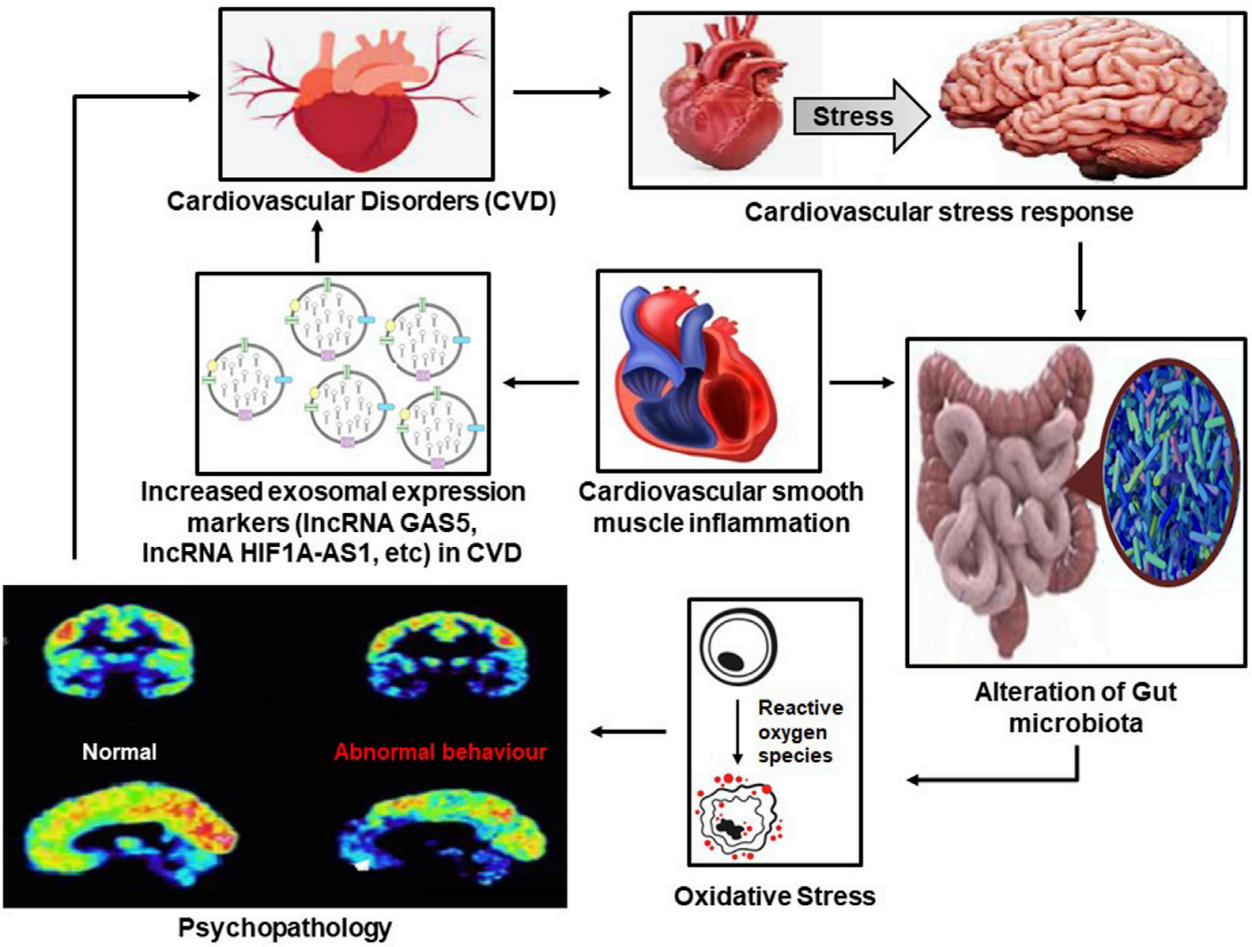
The vicious cycle between psychopathology and cardiovascular diseases (CVD) in which increased exosomal activity and psychopathology result in CVDs, which in turn results in adverse psychopathological outcomes through the alteration of the gut microbiome.

Exploring the nature of the gut-immune axis and neuro-endocrinological status via exosomes may lead to an understanding of the inflammatory responses that can cause cellular damage, resulting in cardiovascular progression. This progressive nature through literature has shown overlapping circumstances in which type II diabetics are impacted by behavioral and psychosocial changes. This is further an apprehension to the contributing effects of cellular functioning and the role of exosomes in their capacity to affect extracellular vesicles. This influence on inflammatory responses and neuronal stress reactions enables an understanding of the gut-brain immune axis with cardiovascular sequelae, which allows a comprehension of the role of these circumstances that emphasizes two primary interventional objectives: 1) preventive measures for addressing and managing stress and exosomes, and 2) intervention methods with the use of stress management can help address the immune and inflammatory responses through exosomes (Figure 2).

Stress has a key role in one’s physiological and psychological manifestations. It acts as a critical role player when it comes to health hierarchies. Psychoneuroendocrine immunology is an emerging concept combining the fields of neuroscience, psychobiology, epigenetics, and molecular biology that aims to identify the effect of stress on human health, highlighting the interaction between the psyche, brain, and body system. Furthering this particular theory, research has identified the role of the interaction between the social and physical environmental influence on the immune system and psychophysical health. According to Bottaccioli et al., (2019), a bidirectional link exists between stress and the immune system, and the same reciprocal interaction has provided evidence for both medical and psychological morbidities.

The cardiovascular stress response is an established marker of lifestyle disease risk. This particular response occurs for several types of stress and has a more pronounced effect when it comes to the biopsychosocial model of the disease. Theoretical models of personality have been linked to the disease based on the adjustment to stress. Trait-based theories of personality have emphasized that type A and D personalities are conceptualized to depict behavior patterns that are prevalent in CVD patients. This further acknowledges the effect of factors such as single stress exposure, adapting to consecutive stress exposures, and responding to the same stressor in different contexts, such as traits and social and biological factors (habituation). This leads to a new approach to perceiving cardiovascular diseases, relating to occurrence, risk, and recovery (Hughes et al., 2018).

### 3.1 Psycho-neuro-endocrinological Correlates in CVD

Extracellular vesicles and membrane-bound organelles contain many bioactive molecules in the form of protein lipids, which tend to influence exosomal production in the extracellular bodies in extracellular space upon the fusion of multivesicular bodies. Which explains the existence of a major link to exosome function, specificaly in cardiac progenitor cell-derived exosomes. This determines the effectiveness of protecting cardiomyocytes against oxidative stress-related apoptosis via exosomal miR-2. Making it a primary trigger point which is a role of exosomal nature and its condition, with an outcome that may be of significance in cardiovascular health (Gao et al., 2019). Understanding this concept lays foundational evidence for the exosomal role in cardiovascular functions and their derivatives.

It has been identified that exosomes (lncRNA GAS5, lncRNA HIF1A-AS1, etc.) derived from cardiomyocytes, endothelial cells, and stem cells of cardiovascular cells can act as vehicles for cytokines, growth factors, and miRNAs, which enables the participation of cardioprotection and post-injury regeneration for the tissues in CVD (Zamani et al., 2019). This explains the regenerative role of exosomes in CVD and further contributes to the understanding that controlling exosomal behavior to progress positive outcomes in cardiovascular diseases has been a progressing effort in addressing cardiovascular diseases outcomes (Guo et al., 2020). This fundamentally establishes exosomal behavior as an excitation factor in CVD care and prognosis.

A similar association of exosomal behavior has been noted with the development of diabetes and its contribution to cardiovascular problems. Diabetes promotes the increased production of miR-221/222 for the exosomes derived from vascular smooth muscle cells that promote atherosclerotic plaque development by increasing endothelial activation and the inflammatory polarization of macrophages (Yu et al., 2023). Additionally, it has been explained that some miRNAs in correspondence to exosomes exploited as targets currently include miR-27a, miR-155, miR-143/145, and miR-16 (associated with a better miR-222), as well as miR-146a, miR-25-3r, miR-16-5r, which are connected to diabetes mellitus type 1, miR-455, miR-296 (related to diabetes mellitus type 2), miR-323-5p, and miR-466. Furthermore, these constructs have been associated with exosomal miRNAs and lncRNAs as they have been shown to play roles in modulating the progression of diabetes, including affecting metabolic and insulin signals in target tissues, cell viability, and pancreatic cell inflammation (Sufianov et al., 2023). This can further indirectly extend to the proposed role of gut-based microbial/microbiome studies that have identified common traits in CVD, in particular, a decreased abundance of gut microbes with a capacity for producing butyrate (Trøseid et al., 2020). Butyrate is predicted to modulate immune and other cells via the regulation of the content of exosomes from intestinal epithelial cells (Anderson, 2019), thus emphasizing the association between exosomal behavior, gut behavior, CVD, and diabetes (Table 1).

**TABLE 1:** Summary of exosomes linked to CVD through the psycho-neuro-endocrinology perspectives discussed.

Exosomes	Relationship to CVD	References
lncRNA GAS5	Increased in the atherosclerotic plaque	Zamani et al. (2019)
lncRNA HIF1A-AS1	The level of exosomal lncRNA HIF1A-AS1 is remarkably higher in patients with atherosclerosis. Hence, it could be a potential biomarker	Zamani et al. (2019)
miR-27a, miR-155, miR-143/145, miR-16 (associated with a better miR-222), miR-146a, miR-25-3r, miR-16-5r	Connected to diabetes mellitus type 1	Sufianov et al. (2023)
miR-455, miR-296	Related to diabetes mellitus type 2	Sufianov et al. (2023)

The gut-brain axis has also been identified to have a bidirectional connection via neural, immune, and endocrine pathways (Asadi et al., 2022). Differences in the microbiota with the suggested role of “microbiota-gut-brain axis” is bidirectional with the gut-brain interactions involving signaling molecules, immune mediators, and gut hormones, where their metabolitesare noted to directly influence the blood, cranial nerves, and central nervous system (Tonomura et al., 2020). The identified relationship of psychological distress is a key negative influencer of significance in various diseases, creating a variety of stress-induced hemodynamic changes that inhibit cellular immune responses producing cytotoxicity and T-12 responses. The same faction creates myocardial ischemia and ventricular arrhythmia in patients with coronary artery disease. This particular association establishes a relationship between psychological distress/stress associated with oxidative stress, clearly suggesting that psychological stress increases oxidant production and oxidative damage, which, upon long-term exposure, increases the risk of developing stress-induced pathophysiology, leading to sub-healthy conditions (Kruk et al., 2019; Wang et al., 2007).

Gut microbiota and stress interaction have been shown to have an inflammatory response in the vascular cells, leading to several cardiovascular conditions and comorbid physiological responses (Rook et al., 2004). Patients with cardiovascular conditions are known to be prone to anxiety and depression. Analyses have shown that the interaction between the gut microbiota and stress could become a leading factor in determining the stress to the CVD pathway (Foxx et al., 2020).

Factors influencing the cardiac conditions and the associative physiological response concerning the psychological constraints are highly interlinked and tend to form a vicious cycle as the recent evidence indicates that cardiac diseases and the medical interventions designed to treat them appear to have serious implications for psychological health, which in turn seem to affect long-term physical health outcomes. Stress is a common factor of relapse in patients with CVD and is known to increase complications. Additionally, cardiac disease and the medical procedures involved in its treatment have been linked to excessive anxiety and worry that may manifest as panic disorder, acute stress disorder, generalized anxiety disorder, and adjustment disorder with anxious mood (Celano et al., 2016).

## 4 Psychological derivatives and CVD

**FIGURE 3:**
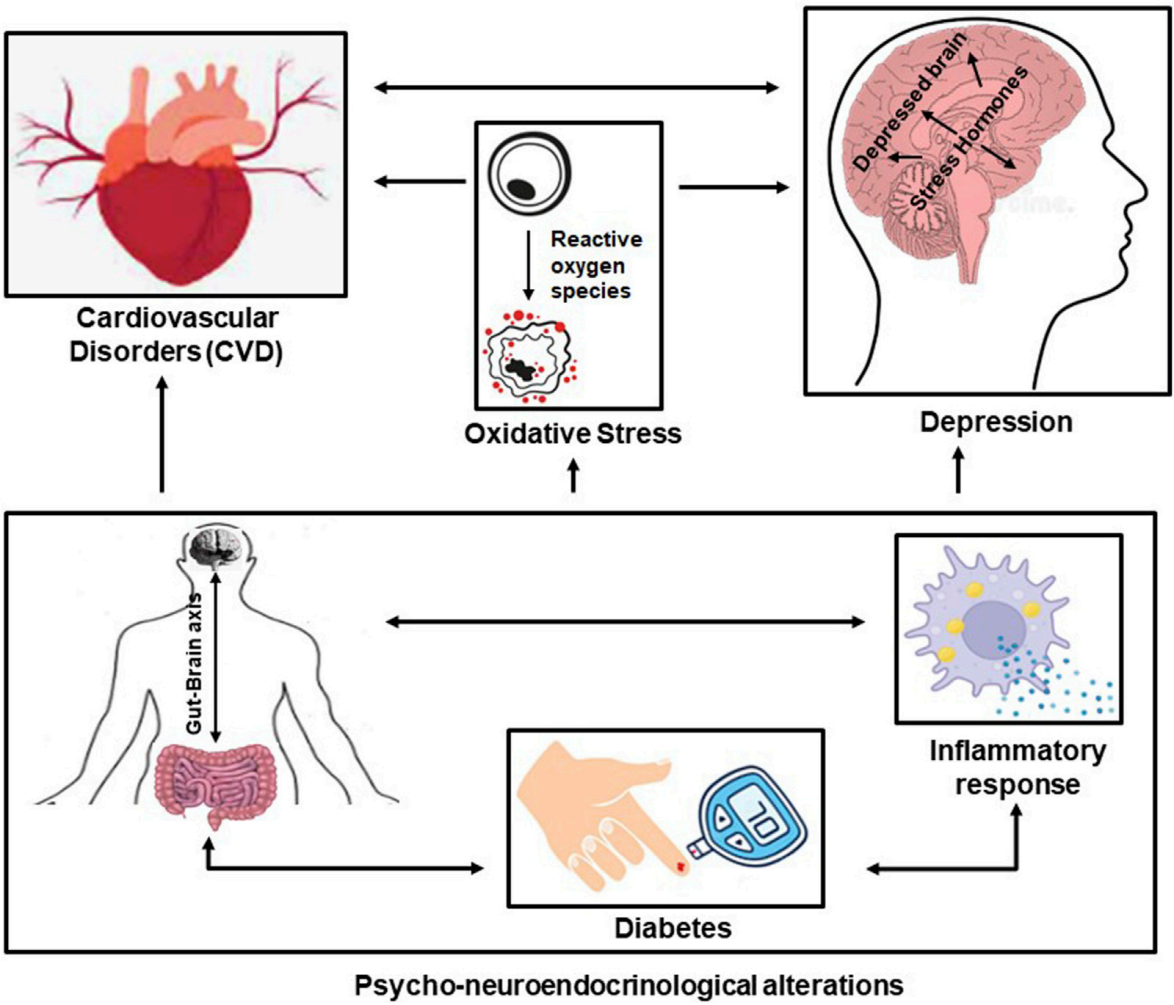
The interlinkage between CVD and depression and the influence of common risk factors (the gut-brain axis, diabetes, and the inflammatory response)—the psycho-neuro-endocrinological alterations—and the influence of oxidative stress.

Several studies have shown that depression and CVD are highly interlinked. Additionally, they have common risk factors, such as obesity and diabetes. An imbalance in the gut microbiota might be a potential biomarker of the development of CVD in patients with T2D (Tsai et al., 2021). Depression and CVD have common influencing factors, such as inflammatory response, diabetes, the gut-brain axis, and oxidative stress (Dhar and Barton, 2016). Oxidative stress seems to be at the center of the whole narrative as risk factors associated with oxidative stress seem to influence and aggregate these diseases, resulting in overreactive oxidative stress and a reduction in the production of antioxidants (Figure 3). Oxidative stress has been observed to increase the risk of depression in CVD patients and the potential for heart conditions in patients with depression (Lin et al., 2019).

Oxidative stress can independently and directly be a causal factor in chronic heart disease and stroke, furthering depression. Cerebral ischemia induction in the brain can promote oxidative stress activity, resulting in DNA damage and molecular peroxidation. Depression is counter-related to CHD and *vice versa* alongside stroke and additionally shares common risk factors, such as obesity, diabetes, and hypertension. The underlying mechanisms involved in many pathological processes, such as inflammation, cell death (apoptosis and autophagy), the microbiome-gut-brain axis, and oxidative stress, also participate in these diseases (Dahlgaard et al., 2019). This reflects on the fact that psychological health is specific to depression and its relationship to how exosomal behavior is associated with CVD, diabetes, and the gut-brain axis, which is collectively governed by oxidative stress, bringing into focus the associated idea of psycho-neuro-endocrinological perturbation. Given the paradigm, the relationship between the gut microbiome, immunological responses, and diabetic behavior influenced by exosomal activities indicates an expected effect that is the main cause of CVD through depression and *vice versa* instills the effect of oxidative stress.

## 5 Discussion

This comprehensive understanding leads to an avenue of intervention in CVD relating to both physiological and psychological management. Physiology focuses on curative components of both pharmacological and invasive procedures alongside nutritive and lifestyle factors. When it comes to psychological management, there is a focus on behavioral activation involving potential mindfulness-based intervention mechanisms between prolonged stress and health. This can be facilitated by collectively focusing on neurological, neuroendocrine, immunological, and molecular manifestations of allostatic load and pathophysiological processes which can be corelated with psychopathological manifestations in the form of behaviour and cognition. Thus, identifying our approaches to addressing CVD for better health invovles facotors associted with it being a lifestyle-based disorder that has a potential for high-risk behavior which can manifest both physiologically and psychologically.

In our attempt to understand the psycho-neuro-endocrinological entities of cardiovascular disorders, the narrative focused on exosomal behavior, the gut-brain axis, immunology, diabetics, and oxidative stress. This Review has summarized the underlying role of exosome cycling in immune functions that can further faciltate the interaction of the gut-brain axis, diabetic risk factors, and the effect of oxidative stress, which collectively reflect on CVD and instigate depressive pathology. Present avenues that can be investigated are the reverse role of psychological interventions in exosomal behaviors validating effective changes in immunology, the gut-brain axis, and oxidative stress. This can significantly change the approach to clinical care in invasive and non-invasive procedures and the effective role of the mind and body hypothesis.
